# Mortality among pediatric patients on HIV treatment in sub-Saharan African countries: a systematic review and meta-analysis

**DOI:** 10.1186/s12889-019-6482-1

**Published:** 2019-02-04

**Authors:** Ismael Ahmed, Seblewengel Lemma

**Affiliations:** 1 University of Gondar, Gondar, Ethiopia; 2 London School of Hygiene and Tropical Medicine, London, UK

**Keywords:** Mortality, Children, Pediatric, Sub-Saharan Africa, Systematic review, Meta-analysis

## Abstract

**Background:**

Despite substantial improvements in accessibility of Anti-Retroviral Treatment (ART), death of children on ART remains a prevailing challenge in sub-Saharan African (SSA) countries. However, the pooled magnitude of mortality at different ART follow-up periods remains unknown for the region. We estimated the pooled proportion of all-cause mortality for pediatric patients receiving first-line ART at 3, 6, 12, and 24 months follow-up period in SSA.

**Methods:**

We searched for relevant articles published between January 2014 and June 2018 on PubMed, Hinari and Google scholar databases. We searched for additional articles from reference lists and 2014–2018 abstracts archived by the Conference on Retroviruses and Opportunistic Infections (CROI) and the International AIDS Society Conference on HIV Science (IAS).

**Results:**

We reviewed 29 articles reporting mortality among pediatric ART patients at different follow-up periods in countries from 2001 to 2016. Among the 51,619 pediatric ART patients in these cohorts, studies reported 4061 (7.9%) all-cause cumulative death. The cumulative pooled proportion of mortality at 3, 6, 12 and 24 months of ART were 3% (95% CI: 3.0–4.0), 5% (95% CI: 4.0–6.0), 6% (95% CI: 5.0–7.0) and 7% (95% CI: 6.0–8.0), respectively.

**Conclusions:**

In SSA, significant proportion of mortality among children occurs in the first 3–6 months of ART initiation. Western Africa has a little higher estimate of mortality among pediatric ART patients at 6 and 12 months of follow-up. Strategies to prevent early mortality including thorough screening and management of opportunistic infections before ART initiation are needed.

**Electronic supplementary material:**

The online version of this article (10.1186/s12889-019-6482-1) contains supplementary material, which is available to authorized users.

## Background

There have been several significant developments in the Human Immunodeficiency Virus (HIV) field since 2013. Evidence revealed that early ART initiation among children results in reduced HIV-associated morbidity and mortality [1, 2]. In line with this evidence the World Health Organization (WHO) revised its recommendation in 2013 to initiate ART for all < 5 years pediatric HIV-infected patients regardless of CD4 cell count or WHO clinical stage [3]. Based on review of additional new evidence [4, 5], this recommendation was expanded in 2016 to “test and treat” all PLHIV [6]. These progressive recommendations were geared towards achieving the Joint United Nations Programme on HIV/AIDS (UNAIDS) 90:90:90 ambitious goal of controlling the Acquired Immune Deficiency syndrome (AIDS) epidemic by 2020 where 90% of all PLHIV will know their HIV status, 90% of all people with diagnosed HIV infection will receive ART, and 90% of all people receiving ART will have viral suppression [7].

Despite substantial improvements in accessibility of ART and improved program implementation, death and loss to follow-up (LTFU) have been a prevailing challenge among PLHIV of all ages. However, attrition is much more pronounced in pediatric cases. There have been various studies conducted in SSA countries to determine the rate of mortality among pediatric ART patients. A systematic review conducted by Fox et al., estimated attrition (death and LTFU) of pediatric ART patients in low- and middle-income countries (LMICs) based on studies from 2008 to 2013 [8]. However, the pooled magnitude of mortality at different ART follow-up periods have not been separately analyzed and reported. The aim of this review is to determine the pooled magnitude of mortality at different follow-up period among pediatric patients who are on first-line ART in SSA countries based on studies published since 2014. This timeframe was selected to include new studies that were not covered in the systematic review conducted in LMICs [8]. The lessons from such studies can guide pediatric HIV program implementation in SSA and help policy makers and program managers to make informed decisions to prevent deaths among pediatric ART patients.

## Methods

### Data sources and searches

We followed the Preferred Reporting Items for Systematic Reviews and Meta-analyses (PRISMA) guidelines for this review [9]. We searched PubMed, Hinari and Google scholar databases for peer-reviewed articles published from January 1, 2014 to June 24, 2018. Medical Subject Headings (MeSH) and free-text search terms used for the search included “mortality”, “death”, “survival”, “retention”, “attrition”, “outcome”, “antiretroviral”, “HIV treatment”, “pediatric”, “paediatric”, “child”, “children”, and “Africa”. We searched conference abstracts from CROI archives using “mortality” and IAS using “mortality” or “survival” from 2014 to 2018. See our exact search strategy used for different databases on Additional file 1. Furthermore, the data source was supplemented by hand searching through reference lists of included studies identified through the search. We limited the search to human subject studies and articles published in English. The review protocol was registered at the PROSPERO international prospective register of systematic reviews (http://www.crd.york.ac.uk/PROSPERO/display_record.php?ID=CRD42018099618), registration number CRD42018099618).

### Study selection

We included observational studies conducted in SSA countries using cohort (prospective or retrospective) study design to determine mortality. We excluded articles that used clinical trial, case-control, cross-sectional, case reports, case series, reviews, qualitative studies, editorials, commentaries, and letters to editors to avoid some level of methodological heterogeneity. We included studies examining HIV-infected children < 18 years old who received standard first-line ART at any type or level of health facility that followed prevailing national treatment guidelines. Studies addressing both adults and children were included when data provided for children were reported separately.

**Fig. 1:**
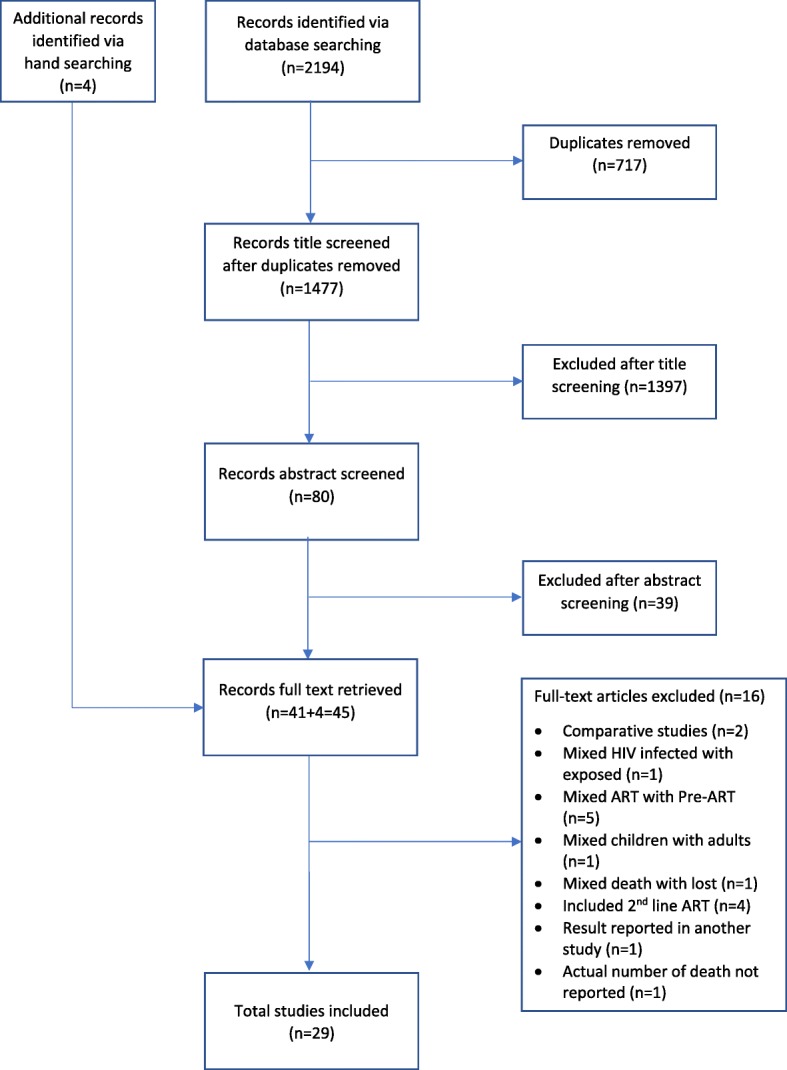
Study selection process and reasons for exclusion of studies

SL supervised the primary search conducted by IA. We obtained full-text copies of all potentially relevant articles after the initial title and abstract screening for further assessment by the two authors based on the eligibility criteria. Full-text articles were reviewed by IA and SL, and any differences were resolved through consensus of both authors. Full-text articles were requested from the authors when they were not available. Articles that were not be eligible at this stage were excluded; and reasons for exclusion were documented (see Fig. 1). Literature obtained using the search strategy were uploaded to Zotero software that helps in collecting, organizing and managing articles.

### Data abstraction

For studies that met the inclusion criteria, relevant data were extracted by the first reviewer (IA) using a predefined Excel tool developed for the review. All extracted data were checked by the second reviewer (SL) to ensure the quality of data abstraction. Any disagreements were resolved by discussion. Relevant variables extracted from studies include name of first author, year of publication, country/setting, year(s) of data collection, sample size, number of facilities, duration of follow-up, sociodemographic characteristics of population (mean or median age, and sex), number of deaths and LTFU at different time period, and incidence of mortality. Where there was missing data or uncertainty about the data, efforts were made to contact study authors for raw data and/or provide clarification.

### Outcomes

The proportion of children who died while taking ART at 3, 6, 12 and 24 months of follow-up period was the primary outcome.

### Quality assessment

We assessed the quality of each of the included studies using the Quality Assessment Tool for Observational Cohort and Cross-sectional Studies provided by the U.S Department of Health and Human Services [10]. The tool has 14 questions or criteria on (1) research question; (2 & 3) study population; (4) groups recruited from the same population and uniform eligibility criteria; (5) sample size justification; (6) exposure assessed prior to outcome measurement; (7) sufficient timeframe to see an effect; (8) different levels of the exposure of interest; (9) exposure measures and assessment; (10) repeated exposure assessment; (11) outcome measures; (12) blinding of outcome assessors; (13) follow-up rate; and (14) statistical analyses. Two authors (IA and SL) independently assessed the studies and any disagreements were resolved by discussion. The final assessment score is given as “good” or “fair” or “poor”.

### Data analysis

We undertook an initial descriptive analysis of all included studies that have reported aggregate all-cause mortality among children enrolled in ART. Meta-analysis was performed to estimate the pooled mortality and its 95% confidence interval (CI) for cohorts that reported number of children died at different follow-up periods. We interpolated or extrapolated data for any missing time period when possible for a few (*n* = 4) of the studies [11–14]. For example, if a cohort reported 6- and 24-months mortality, we interpolated 12 months mortality assuming a linear increase between two points. Heterogeneity between studies was examined using the I^2^ statistic and the *p*-value for heterogeneity [15]. I^2^ ≥ 75% or *P* < 0.1 was considered as high statistical heterogeneity [16]. We plotted each mortality estimate and its 95% CI using forest plots and combined estimates using a random-effects model after stabilizing the variance by transforming the proportions using the Freeman-Tukey double-arcsine method because of evidence of high heterogeneity between studies [17].

Sub-group analysis of pooled mortality at 3, 6, 12 and 24 months was conducted. Countries were divided geographically into three groups as Eastern, Western and Southern Africa. We have excluded multinational studies that reported combined results of Southern and Eastern African countries. Accordingly, a study conducted by Ben-Farhat et al. [11] was excluded from the 3, 6 and 24 months pooled mortality analysis. Similarly, both Ben-Farhat et al. [11] and Lamb et al. [18] were excluded from the 12 months pooled mortality analysis. All meta-analysis results were summarized graphically using Forest plots. Statistical analyses were performed using STATA version 12.

### Sensitivity analysis

To determine the proportion of the summary results driven by some studies, we conducted three different sensitivity analysis by excluding studies with the largest sample size [19, 20], studies with high rate of LTFU (> 15%) [20–23], and studies with missing data (without interpolation or extrapolation) [11–14].

### Publication bias

We used “metabias” that performs the Begg and Mazumdar adjusted rank correlation test and the Egger et al. regression asymmetry test for publication bias when ten or more studies reported mortality at different time periods. Failure of this confidence interval to include zero or *p*-value < 0.1 indicates asymmetry in the funnel plot and may give evidence of publication bias [24–26].

## Results

Our search strategy returned a total of 2194 articles, and 1477 remained after de-duplication. Of the 1477 articles, 41 studies were eligible for full-text review. Four additional studies were hand searched and included for full-text review. After screening full-text of 45 studies with our inclusion criteria, an additional 16 studies were excluded because of various reasons indicated in Fig. 1. In total, 29 cohort studies (journal articles) were selected for inclusion in the review. Searching for grey literature using conference abstracts archives resulted in one eligible abstract which was rejected later due to inaccessible full-text for final review. Of 22 study authors contacted for raw data/or provide clarification, 50% responded to our request. All of the included studies, except three [27–29] were rated as “good” for the methodological quality assessment. See Additional file 2 for detailed quality assessment results of all studies.

### Study characteristics

The characteristics of studies included in the review are summarized in Table 1. The studies were published between 2014 and 2018, but studied all-cause mortality among cohorts of children enrolled on ART from 2001 to 2016 in 15 SSA countries. One third (*n* = 5, 33%) of the countries had four or more studies. The highest number of publications were from Ethiopia (*n* = 9) followed by Malawi (n = 5) and South Africa (n = 5). There were five multi-national studies [11, 18, 19, 21, 30]. The total sample size of the combined studies was 51,619, with each cohort ranging from 93 to 10,875 children. About 90% (*n* = 26) of the studies reported deaths among children 15 years of age or younger [11–13, 18–22, 27–44]. Nineteen studies reported median or mean age of children which is ranged from 0.5 to 9 years [11–14, 21, 22, 27, 29, 30, 32–36, 41–45].

**Table 1:** Characteristics of studies included in the review (*n* = 29)

Author	Country	Year of publication	Facilities (n)	Facility type	Cohort size (n)	Time of cohort observation	Children’s age	Median or mean age (years)	Female (%)
Abrams [28]	South Africa	2017	5		226	Jan.-Dec. 2011	< 2 years		
Andargie [31]	Ethiopia	2018	1	Public	269	2008–2013	<15 years		56.9
Anigilaje [32]	Nigeria	2018	1	Public	368	Oct. 2010-Dec. 2013	<15 years	5.6	44.0
Auld [34]	Côte d’Ivoire	2014	29		2110	2004–2008	<15 years	5.1	46.0
Auld [33]	Mozambique	2015	25		1054	2004–2009	<15 years	3.3	50.0
Ben-Farhat [11]	Malawi, Uganda and Kenya	2017	4	Public & NGO	3949	Dec. 2001-Dec. 2010	<15 years	4.2	49.6
Biru [35]	Ethiopia	2018	8	Public	304	2014–2016	3 month-14 years	9	48.4
Brophy [22]	Malawi	2016	31	Public	2203	Oct. 2003-Sept. 2011	<15 years	4.8	50.6
Davies [19]	Malawi, South Africa, Zambia and Zimbabwe	2014	11		12,655	2004–2010	< 10 years		
Ditekemena [36]	Democratic Republic of Congo	2014	3	Private & NGO	522	Sept. 2007-May 2012	6 month-14 years	4.7	49.0
Ebissa [12]	Ethiopia	2015	4	Public	556	Jan. 2008-Dec. 2009	1–12 years	6.3	47.5
Ebonyi [13]	Nigeria	2014	1	Public	691	Jul. 2005-Mar. 2013	2 month-15 years	3.7	48.9
Edessa [37]	Ethiopia	2015	2	Public	305	Sept. 2010-Mar. 2013	<15 years		52.1
Kedir [38]	Ethiopia	2014	1	Public	560	Jan. 2006-Dec. 2010	< 14 years		50.6
Lamb [18]	Kenya, Mozambique, Tanzania and Rwanda	2014	160		2045	Jan. 2005-Sept. 2010	10–14 years		
Lilian [29]	South Africa	2017	106		5461	Jan. 2005-Dec. 2014	<15 years	5.0	50.8
Marazzi [30]	Mozambique, Malawi and Guinea	2014	17	Public	2215	Jan. 2005-Dec. 2008	<15 years	4	47.7
McHugh [39]	Zimbabwe	2017	7	Public	296	Jan. 2013-Dec. 2014	6–15 years		
Melaku [20]	Ethiopia	2017	70	Public	6815	Jan. 2006-Sept. 2013	<15 years		
Mokgatle [45]	Ethiopia	2016	1	Public	786	Mar. 2005-Mar. 2012	< 18 years	7.9	49.7
Mulugeta [40]	Ethiopia	2017	4	Public	757	Jan. 2008-Dec. 2010	<15 years		49.0
Naik [41]	Tanzania	2016	2	NGO	93	Mar.-Dec. 2011	< 2 years	1.1	46.2
Njom [14]	Cameroon	2017	1		197	2005–2009	< 17 years	3	46.0
Ojeniran [42]	Nigeria	2015	1		660	2005–2011	<15 years	3.4	49.7
Ojikutu [23]	Nigeria	2014	23		1516	Nov. 2002-Dec. 2011	< 18 years		47.2
Porter [21]	South Africa, Zimbabwe, Malawi and Zambia	2015			4945	Jan. 2004-Dec. 2012	<1 years	0.5	51.5
Sidamo [44]	Ethiopia	2017	2	Public	407	Jan. 2009-Dec. 2016	< 14 years	6	42.8
Teasdale [43]	South Africa	2017	1		681	Jan. 2004-Sept. 2015	<15 years	3	48.5
Vermund [27]	Mozambique	2014			753	Jun. 2006-Jul. 2011	<15 years	1	52.5

Blank spaces mean data is not available or not reported

### Magnitude of mortality

A total of 4061 all-cause cumulative deaths were reported by the 29 included studies that had various cohort follow-up time periods. The highest proportion of deaths was reported by a study in South Africa (21.0%, *n* = 143) [43]; and the lowest was from one of an Ethiopian cohort (2.0%, *n* = 6) [35]. Among 14 studies that reported on follow-up time of children on ART, the median or mean follow-up time ranged from 11 to 68 months for multi-country cohort in South Africa, Zimbabwe, Malawi and Zambia [21] and Ethiopia [40], respectively. Incidence of mortality was reported by 13 studies and ranged from 0.98 to 6.9 per 100-child year in Mozambique [33] and among a multi-country cohort from Mozambique, Malawi and Guinea [30], respectively.

Among studies that reported on mortality along with the patients’ follow-up period, proportion of mortality at 3 months of ART ranged between 1.1 and 7.8% in cohorts from Tanzania [41] and Mozambique [27], respectively. Proportion of mortality at 6 months ranged between 1.6% in cohorts from Ethiopia [35] and 12.4% in cohorts from South Africa [28]. The South African study focused on younger children < 2 years of age. Proportion of mortality at 12 months ranged between 2.0% in cohorts from Ethiopia [35] and 16.1% in cohorts from Mozambique [27]. Similarly, at 24 months of ART, proportion of mortality ranged from 3.8% among cohorts in Nigeria [23] to 20.2% among cohorts from Mozambique [27]. Table 2 shows proportion of deaths at each reported time period by country.

**Table 2:** Follow-up time and proportion of mortality reported on studies included in the review (n = 29)

Author	Country	Patients started on ART (N)	Median or mean follow-up years	Child-years of follow-up	Overall incidence of death/100 PY	Total reported death		Proportion of death (%)			
						n	%	3 months	6 months	12 months	24 months
Abrams [28]	South Africa	226				28	12.4		12.4		
Andargie [31]	Ethiopia	269				46	17.1				
Anigilaje [32]	Nigeria	368			3.0	40	10.9		8.2	2.7	
Auld [34]	Côte d’Ivoire	2110		4585	5.17	237	11.2	6.4			
Auld [33]	Mozambique	1054		2652	0.98	26	2.5				
Ben-Farhat [11]	Malawi, Uganda and Kenya	3949	2	5858	5.1	299	7.6	3.4	4.9	5.8^a^	7.6
Biru [35]	Ethiopia	304	0.99	287.7	2.1	6	2.0		1.6^c^	2.0	
Brophy [22]	Malawi	2203	1.5	3900	3.4	134	6.1			4.8	
Davies [19]	Malawi, South Africa, Zambia and Zimbabwe	12,655				877	6.9			6.9	
Ditekemena [36]	Democratic Republic of Congo	522	3			22	4.2				
Ebissa [12]	Ethiopia	556				58	10.4		7.4	8.5^a^	10.4
Ebonyi [13]	Nigeria	691	4.4	2752	1.0	32	4.6		3.8	3.9	4.2^a^
Edessa [37]	Ethiopia	305		609	3.2	28	9.2	4.3	5.9	7.2	9.2
Kedir [38]	Ethiopia	560	3.9	2078	2.06	43	7.7	4.1	5.9	7.0	7.1
Lamb [18]	Kenya, Mozambique, Tanzania and Rwanda	2045				91	4.4			4.4	
Lilian [29]	South Africa	5461	2.2			300	5.5		3.1	3.8	4.4
Marazzi [30]	Mozambique, Malawi and Guinea	2215	1.5		6.9	238	10.7				
McHugh [39]	Zimbabwe	296	1.3		2.86	12	4.1				
Melaku [20]	Ethiopia	6815				327^b^	4.8^b^		3.4^b^	4.1^b^	4.8^b^
Mokgatle [45]	Ethiopia	786	3.4		2.3	62	7.9	4.2			
Mulugeta [40]	Ethiopia	757	5.7	4112	1.24	51	6.7	2.2	3.6	4.8	5.5
Naik [41]	Tanzania	93				4	4.3	1.1	2.2	4.3	
Njom [14]	Cameroon	197	4			20	10.2		7.1	7.6^a^	8.6^a^
Ojeniran [42]	Nigeria	660		2468.7		66	10.0				
Ojikutu [23]	Nigeria	1516	2.3			64	4.2	2.0	2.4	3.2	3.8
Porter [21]	South Africa, Zimbabwe, Malawi and Zambia	4945	0.93			596	12.1		8.7	10.7	
Sidamo [44]	Ethiopia	407				59	14.5		3.4	4.4	4.9
Teasdale [43]	South Africa	681				143	21.0				
Vermund [27]	Mozambique	753				152	20.2	7.8	12.0	16.1	20.2

^a^Interpolated/extrapolated data. ^b^Data extracted from cumulative incidence ^c^Six months data is at 8 months. Blank spaces mean data is not available or not reported

### Meta-analysis of mortality

Meta-analysis of pooled estimates of mortality were conducted for studies that have reported on proportion of children who died at different follow-up periods. We excluded studies with poor methodological quality [27–29]. Accordingly, a total of 8 [11, 23, 34, 37, 38, 40, 41, 45], 14 [11–14, 20, 21, 23, 32, 35, 37, 38, 40, 41, 44], 17 [11–14, 18–23, 32, 35, 37, 38, 40, 41, 44] and 10 [11–14, 20, 23, 37, 38, 40, 44] studies were included in the meta-analysis to estimate pooled mortality at 3, 6, 12 and 24 months of follow-up period, respectively. The overall random-effects cumulative pooled proportion of mortality at 3, 6, 12 and 24 month of ART initiation were 3.0% (95% CI: 2.0–5.0), 5.0% (95% CI: 3.0–6.0), 6.0% (95% CI: 5.0–7.0) and 6.0% (95% CI: 5.0–8.0), respectively. The between-study heterogeneity was significant (*p*-value < 0.0001), with I^2^ value of 88.11% for 3 months, 93.99% for 6 months, 95.32% for 12 months, and 88.44% for 24 months.

### Sub-group analysis

Sub-regional analysis of pooled mortality at 3, 6, 12 and 24 months indicated that, the pooled proportion of mortality at 3 months of ART initiation were 4.0% (95% CI: 4.0–5.0) in Western and 3.0% (95% CI: 2.0–4.0) in Eastern Africa regions. The 6 months pooled estimate were 5.0% (95% CI: 3.0–8.0) in Western and 4.0% (95% CI: 3.0–5.0) in Eastern Africa regions. The 12 months pooled estimate were 6.0% (95% CI: 3.0–10.0) in Western, 5.0% (95% CI: 4.0–7.0) in Eastern, and 8.0% (95% CI: 5.0–11.0) in Southern Africa regions. Similarly, the 24 months pooled estimate were 5.0% (95% CI: 3.0–7.0) in Western and 7.0% (95% CI: 5.0–9.0) in Eastern Africa regions. In all the meta-analyses, the between-study heterogeneity was significant (> 75%) with p-value of < 0.001 except for few studies reported from West African at 3 and 24 months.

### Sensitivity analysis

To determine what proportion of the summary results were driven by studies with the largest study population, we conducted a sensitivity analysis by excluding Melaku et al. [20] for the 6, 12 and 24 months estimate and both Melaku et al. [20] and Davies et al. [19] for the 12 month estimate (more than 40% of all the meta-analysis study participants). Following removal of these studies, the overall pooled estimate at 24 months increased by 1% (7.0% (95% CI: 5.0–8.0)).

In order to examine the impact of extrapolated or interpolated data, we conducted sensitivity analysis by excluding Ben-Farhat et al. [11] and Ebissa et al. [12] for 12 months estimate and Ebonyi et al. [13] and Njom et al. [14] for the 24 months estimate which had missing data. Following removal of these studies, the overall pooled estimate was not changed for the different follow-up periods indicating that our results were not driven by studies with extrapolated/interpolated data.

**Fig. 2:**
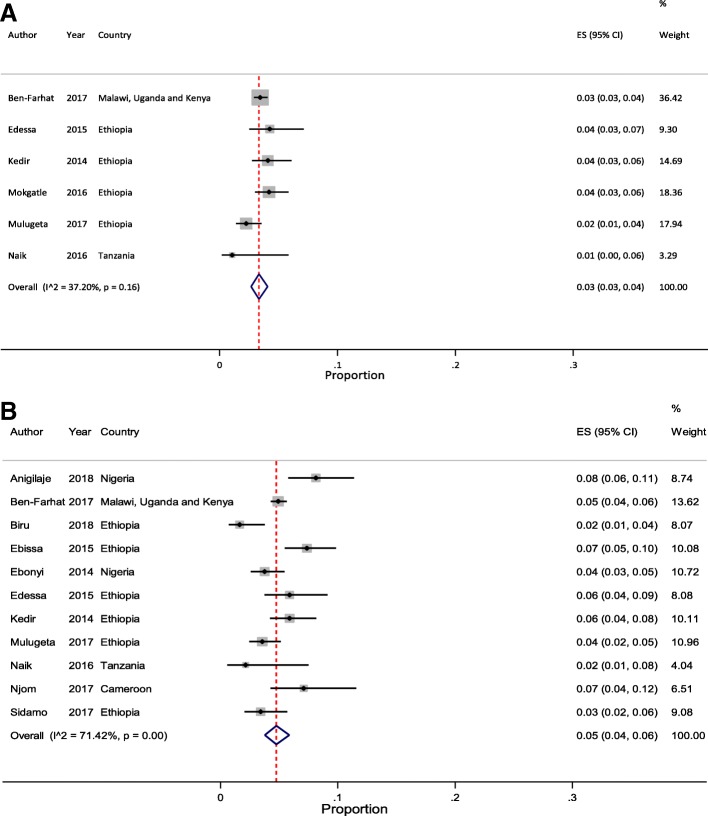
Forest plot of proportion of children died at 3- and 6-months of ART follow-up. **a** Studies reporting to 3 months ART follow-up (n = 6). **b** Studies reporting to 6 months ART follow-up (*n* = 11)

**Fig. 3:**
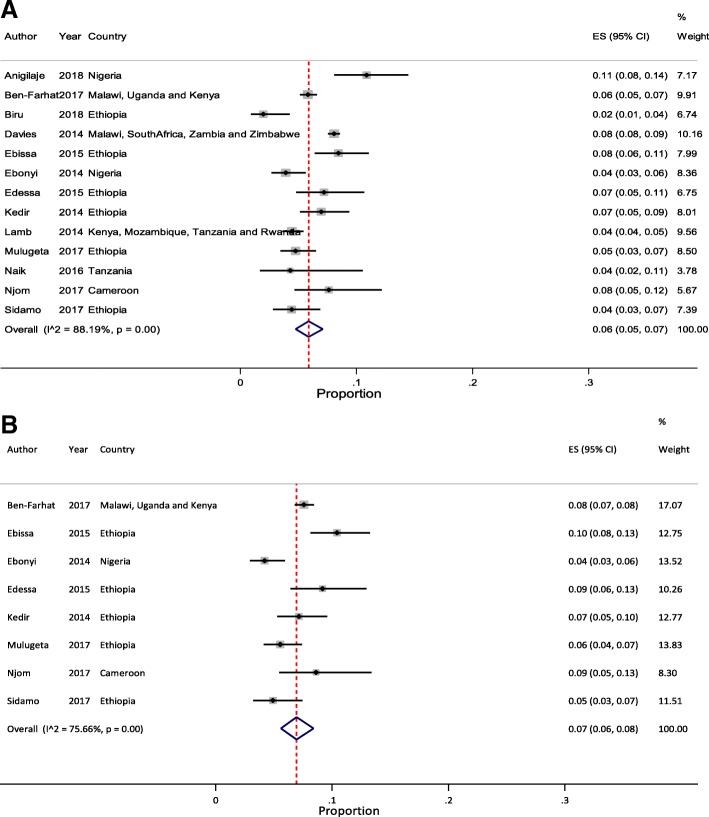
Forest plot of proportion of children died at 12- and 24-months of ART follow-up. **a** Studies reporting to 12 months ART follow-up (*n* = 13). **b** Studies reporting to 24 months ART follow-up (*n* = 8)

In addition, to determine the effect of studies with high rate of LTFU (> 15%), we conducted sensitivity analysis by excluding Auld et al. [34] and Ojikutu et al. [23] for 3 months estimate; Melaku et al. [20], Ojikutu et al. [23], and Porter et al. [21] for 6 months estimate; Brophy et al. [22], Melaku et al. [20], Ojikutu et al. [23], and Porter et al. [21] for 12 months estimate; and Melaku et al. [20] and Ojikutu et al. [23] for 24 months estimate. Following removal of these studies, the pooled estimate was not changed except for the 24 months estimate (7.0% (95% CI: 6.0–8.0)) which increased by 1%, compared with the original finding. However, excluding these studies has lowered the heterogeneity between-studies. We reported this result as the pooled estimates of mortality among pediatric patients on HIV treatment in SSA. Figure 2a and b illustrate mortality rates at 3 and 6 months and Fig. 3a and b illustrate mortality rates at 12 and 24 months of ART initiation with 95% CI and I^2^ using forest plots.

**Fig. 4:**
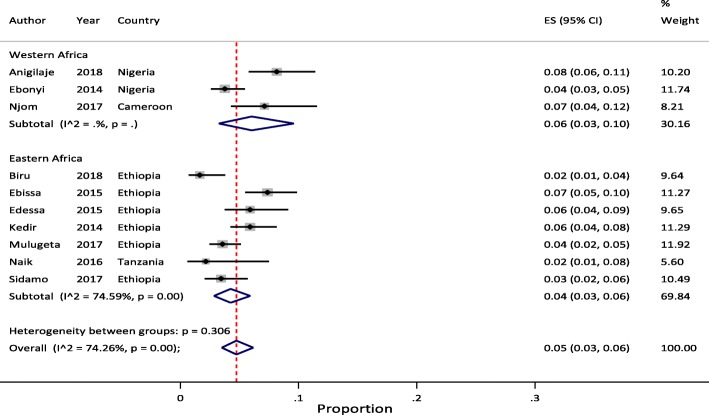
Forest plot of proportion of children died at 6 months of ART follow-up by sub-regions

**Fig. 5:**
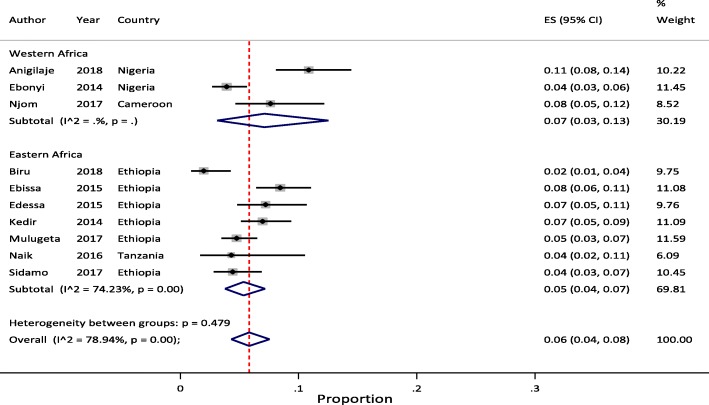
Forest plot of proportion of children died at 12 months of ART follow-up by sub-regions

**Fig. 6:**
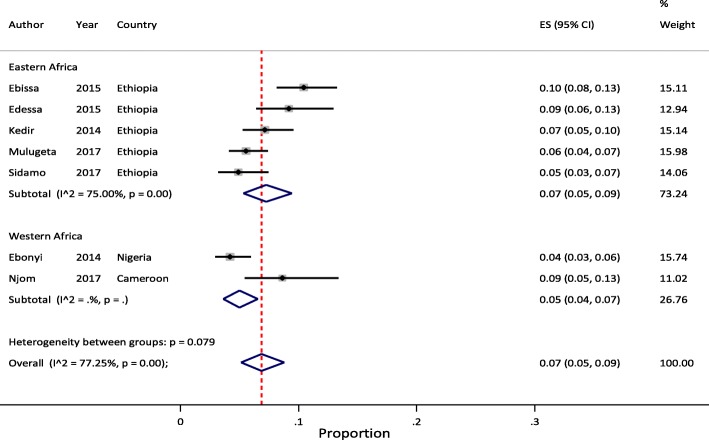
Forest plot of proportion of children died at 24 months of ART follow-up by sub-regions

Considering the above effect of LTFU on heterogeneity of studies, we conducted sub-regional analysis by omitting studies with high rate of LTFU [20–23]. After excluding these studies, the pooled estimate was not changed except for 6 months (6.0% (95% CI: 3.0–10.0)) and 12 months (7.0% (95% CI: 3.0–13.0)) estimates in Western Africa region which increased by 1%, compared with the original findings. However, excluding these studies has lowered the between-study heterogeneity. We did not report pooled mortality at 3 month of ART initiation by sub-regions due to limited number of studies to compare. Similarly, there were no eligible studies from Southern Africa for this sub-regional analysis except a single report at 12 months follow-up time [19]. We reported this result as the sub-regional pooled estimates of mortality among pediatric patients on HIV treatment in SSA. Figures 4, 5 and 6 illustrate mortality rates by sub-regions at 6, 12 and 24 months of ART initiation with 95% CI and I^2^ using forest plots, respectively.

### Publication bias

The funnel plot and nonsignificant Egger’s test was conducted for all eligible studies included in the original meta-analysis. The test for 14 studies reported 6 months data (*P* > 0.402), 17 studies reported 12 months data (*P* > 0.702), and 10 studies reported 24 months data (*P* > 0.191) show no evidence of asymmetry suggesting no publication bias. There was graphical symmetry of funnel plots (see Additional file 3).

## Discussion

We conducted a systematic review and meta-analysis of mortality of HIV-infected children started on first-line ART at different follow-up time periods in SSA countries. The cohort size for this review included 51,619 pediatric patients on first-line ART in 15 SSA countries reported by studies published between 2014 and 2018. The cumulative pooled estimate of this study suggests that ART programs in SSA have been losing 3.0, 5.0, 6.0, and 7.0% of HIV-infected children due to death at 3, 6, 12 and 24 months after ART initiation, respectively. Nevertheless, these cumulative proportion of death at different time periods were not consistently reported by all the studies. We identified that nine studies reported mortality at 3 months, seventeen studies reported mortality at 6 months, 16 studies reported mortality at 12 months, and ten studies reported mortality at 24 months of ART follow-up.

The review showed some degree of variability on attrition of pediatric cohorts enrolled in ART due to death. The overall proportion of death among pediatric patients enrolled on ART across all cohorts ranged between 2.0% from Ethiopia [35] and 21.0% from South Africa [43]. A systematic review conducted in LMICs reported a higher proportion of mortality, ranging between 0.0% from Botswana and 26.0% from Mozambique [8]. This difference could be due to a variation in ART enrollment periods where the previous studies addressed children who started ART in earlier years (before 2012) with different treatment eligibility criteria. Since 2013, the WHO has made significant changes in the recommended pediatric age for ART initiation, regardless of clinical or immunologic status [3, 6, 46] based on evidence that supported early initiation of ART to decrease childhood HIV-associated morbidity and mortality [1, 2]. However, there was variation among SSA countries in terms of time to adopt and implement new recommendations [47].

There are very few similar reviews against which we can compare our findings of pooled mortality estimates at different time period after ART initiation among pediatric patients. A pooled analysis of individual data from 16 clinics in SSA countries provided estimates of mortality at 6, 12 and 24 months of ART follow-up in pediatric cohorts enrolled until 2007 [48]. Our review reported the same results for all the indicated follow-up periods that reaffirm the previous findings. On the other hand, a systematic review without pooled estimate of mortality conducted to evaluate the effectiveness of pediatric ART in resource-limited settings reported 12 months proportion of mortality ranging from 0.0–18.8% [49] among patient cohorts of 2008 or earlier. A similar review conducted in Africa to describe retention of HIV-infected children in the first year of ART indicated that reported mortality ranging from 3 to 15% among studies published between 2006 and 2013 [50]. These findings were somewhat similar with our report at 12 months post-ART where the mortality was estimated to be between 2.0 and 16.1%.

Sub-regional analysis of pooled estimates of mortality conducted to minimize heterogeneity of studies indicated a little higher estimate in Western Africa compared with Eastern Africa at 6 and 12 months of ART follow-up. This could be due to a difference in study settings and cohort years. For instance, the Eastern African studies included recent cohorts up to 2016 who have benefited from early initiation of ART due to a change in the WHO’s recommendation to start ART for all children under 5 years of age since 2013 [3]. On the other hand, the pooled estimates of mortality in this review showed higher deaths among children within the first 6 months of ART initiation. This finding is in congruent with a systematic review conducted in Africa to estimate the effectiveness of cotrimoxazole prophylaxis and ART in HIV-infected children which reported higher (more than half) number of the deaths during the first 6 months of ART initiation [51]. This could be due to undiagnosed Immune Reconstitution Inflammatory Syndrome (IRIS) which is a common complication in patients starting ART specially among patients with advanced disease stage and low CD4 cell count in African cohorts [52]. Studies conducted in South Africa revealed that 21% of children initiating ART developed IRIS [53]; and that IRIS accounted for one quarter of deaths in the first 6 months [54]. A systematic review conducted in low- and high-income countries reported a higher incidence of IRIS following ART initiation [55].

Our review has some limitations. Firstly, similar to a previous systematic review of retention of pediatric patients in LMICs [8], our review included only documented mortality. Most of the studies indicated that their reported mortality could be underestimated in the absence of a mechanism to ascertain the outcomes among children reported as LTFU. In this review, reported LTFU ranged from 2.5% [19] to 31.9% [27] that could underestimate pooled mortality. It is important for future studies to ascertain outcomes of LTFU pediatric ART patients to estimate the true proportion of deaths in this population. Secondly, our review identified a need for additional information on number of deaths at different follow-up time points. In order to fill the gap, we contacted 22 authors through email but only half provided additional information as requested. Thus, we excluded seven studies that did not report mortality at any of the follow-up times from the pooled estimates; and interpolated or extrapolated missing values for four studies. Thirdly, in our review, since few studies reported on mortality beyond 24 months of ART follow-up, we could not estimate the long-term pooled proportion of children who died while on ART. Fourthly, the review was limited to English language studies and we could have missed studies that were published in other languages. Finally, our review revealed significant heterogeneity among studies. This could be partly explained by a difference in age categories of pediatric patients studied, duration of ART follow-up periods, and ART eligibility criteria due to a change in the WHO’s recommendation among the studies. In addition, during sensitivity analysis, we found that studies with high rate of LTFU (> 15%) have contributed to the significant heterogeneity between-studies. In this review, we reported pooled estimates by excluding these studies to minimize the heterogeneity. The result from excluding these studies did not have much effect on the pooled estimates but significantly reduced the heterogeneity between-studies, compared with the original estimates. Furthermore, we conducted sub-regional analysis of pooled estimates along their geographical setting to further minimize the heterogeneity. Despite this heterogeneity, we believed that pooling the estimates of mortality at different follow-up time will provide a more robust estimate than any single study alone that better inform pediatric ART programs in SSA countries. It also helps policy makers and program managers to make informed decisions to prevent deaths among pediatric ART patients at different follow-up periods. However, the pooled estimates should be interpreted with caution.

This review also has some strengths. First, mortality estimates at different time periods has never been addressed suitably by the available albeit limited systematic reviews conducted on retention or attrition (combining death and LTFU) among children after enrollment in ART. Our study fills this gap by offering mortality estimates at different time periods after initiation on ART to inform pediatric HIV programs in SSA. Second, we have conducted multiple sensitivity analysis to assess the impact of studies with high sample size cohorts, high rate of LTFU, and missing data for any time period on the estimated pooled proportion of mortality at different follow-up periods. Third, we have conducted sub-regional analysis to minimize heterogeneity and provide pooled estimates of mortality by sub-regional settings.

## Conclusions

In conclusion, our review indicated that there is high rate of early childhood mortality in the first 3–6 months of ART initiation in SSA that calls for thorough screening and management of opportunistic infections before ART initiation. The review showed some degree of variability on mortality estimates of pediatric cohorts enrolled in ART in the Eastern and Western parts of SSA. The information gap on the long-term outcome (mortality) of ART among pediatric cohorts in SSA demands future investigation. Efforts to track LTFU should be strengthened to understand the outcome of pediatric ART patients. Immediate ART initiation among children under the current “test and treat” strategy should be accompanied by strategies for early identification of HIV-infected children in SSA.

## Additional files

Additional file 1: Databases used and exact search terms. (DOCX 22 kb)

Additional file 2: Quality assessment results for each cohort study included in the review using Quality Assessment Tool for Observational Cohort and Cross-sectional Studies provided by the U.S Department of Health and Human Services. (DOCX 126 kb)

Additional file 3: Begg’s funnel plots to assess publication bias of studies by reporting duration. A) Studies reporting to 6 months ART follow-up (n = 14). B) Studies reporting to 12 months ART follow-up (*n* = 17). C) Studies reporting to 24 months ART follow-up (*n* = 10). (DOCX 21 kb)

## Abbreviations

AIDS: Acquired Immune Deficiency Syndrome
ART: Antiretroviral Therapy
CROI: Conference on Retroviruses and Opportunistic Infections
HIV: Human Immunodeficiency Virus
LMICs: Low- and middle-income countries
LTFU: Loss to Follow-Up
MeSH: Medical Subject Headings
PLHIV: People Living with HIV
PRISMA: Preferred Reporting Items for Systematic Reviews and Meta-analyses
SSA: Sub-Saharan Africa
UNAIDS: Joint United Nations Programme on HIV/AIDS
WHO: World Health Organization
