# Supplementary material for: Mortality among pediatric patients on HIV treatment in sub-Saharan African countries: a systematic review and meta-analysis
Source: BMC Public Health. 2019 Feb 4;19:149. doi: 10.1186/s12889-019-6482-1 (PMC6360742; doi:10.1186/s12889-019-6482-1)
Supplement: Supplementary file 1 — Databases used and exact search terms. (DOCX 22 kb) [file 12889_2019_6482_MOESM1_ESM.docx]

| Database | Search terms and steps |
| --- | --- |
| PubMed | 1. Search ((((((child mortality[MeSH Terms]) OR mortality) OR death) OR analyses, survival[MeSH Terms]) OR retention[MeSH Terms]) OR attrition) OR outcome 2. Search (((antiretroviral agents[MeSH Terms]) OR antiretroviral therapy, highly active[MeSH Terms]) OR antiretroviral) OR HIV treatment 3. Search #1 AND #2 4. Search (((children[MeSH Terms]) OR child) OR pediatric) OR paediatric 5. Search #3 AND #4 6. Search (africa[MeSH Terms]) OR africa, sub saharan[MeSH Terms] 7. Search #5 AND #6, limits: period (2014-2018); human; English and AIDS |
| Hinari | “mortality” AND “antiretroviral” AND “children” AND “Africa” |
| Google scholar | 1. “mortality” AND “antiretroviral” AND “children” 2. “survival” AND “antiretroviral” AND “children” 3. “death” AND “antiretroviral” AND “children” 4. “outcome” AND “antiretroviral” AND “children” 5. “retention” AND “antiretroviral” AND “children” 6. “attrition” AND “antiretroviral” AND “children” |
| Conference on Retroviruses and Opportunistic Infections (CROI) abstract archive | mortality |
| The International AIDS Society Conference on HIV Science (IAS) | “mortality” OR “survival” |
